# Machine learning segmentation of core and penumbra from acute stroke CT perfusion data

**DOI:** 10.3389/fneur.2023.1098562

**Published:** 2023-02-23

**Authors:** Freda Werdiger, Mark W. Parsons, Milanka Visser, Christopher Levi, Neil Spratt, Tim Kleinig, Longting Lin, Andrew Bivard

**Affiliations:** ^1^Melbourne Brain Centre, Department of Neurology, The Royal Melbourne Hospital, Melbourne, VIC, Australia; ^2^Department of Medicine, University of Melbourne, Melbourne, VIC, Australia; ^3^Southwestern Sydney Clinical School, University of New South Wales, Sydney, NSW, Australia; ^4^Department of Neurology, Liverpool Hospital, Liverpool, NSW, Australia; ^5^Ingham Institute for Applied Medical Research, Liverpool, NSW, Australia; ^6^Hunter Medical Research Institution, University of Newcastle, Newcastle, NSW, Australia; ^7^Department of Neurology, John Hunter Hospital, University of Newcastle, Newcastle, NSW, Australia; ^8^Department of Neurology, Royal Adelaide Hospital, Adelaide, SA, Australia

**Keywords:** acute ischemic stroke, CT perfusion imaging, machine learning, ischemic core, penumbra

## Abstract

**Introduction:**

Computed tomography perfusion (CTP) imaging is widely used in cases of suspected acute ischemic stroke to positively identify ischemia and assess suitability for treatment through identification of reversible and irreversible tissue injury. Traditionally, this has been done *via* setting single perfusion thresholds on two or four CTP parameter maps. We present an alternative model for the estimation of tissue fate using multiple perfusion measures simultaneously.

**Methods:**

We used machine learning (ML) models based on four different algorithms, combining four CTP measures (cerebral blood flow, cerebral blood volume, mean transit time and delay time) plus 3D-neighborhood (patch) analysis to predict the acute ischemic core and perfusion lesion volumes. The model was developed using 86 patient images, and then tested further on 22 images.

**Results:**

XGBoost was the highest-performing algorithm. With standard threshold-based core and penumbra measures as the reference, the model demonstrated moderate agreement in segmenting core and penumbra on test images. Dice similarity coefficients for core and penumbra were 0.38 ± 0.26 and 0.50 ± 0.21, respectively, demonstrating moderate agreement. Skull-related image artefacts contributed to lower accuracy.

**Discussion:**

Further development may enable us to move beyond the current overly simplistic core and penumbra definitions using single thresholds where a single error or artefact may lead to substantial error.

## 1. Introduction

Rapid diagnosis of acute ischemic stroke is of vital importance and is confirmed by computed tomography (CT) or magnetic resonance (MR) imaging. Historically improved patient outcomes were obtained by early reperfusion treatment, with significant effort and resources being provided to improve both stroke detection and clinical workflows to facilitate faster treatment (1–3). Recently, clinical trials have demonstrated that patients with a favorable perfusion imaging profile benefit from treatment up to 9 h from symptom onset/mid-point of wake-up with thrombolysis and up to 24 h with thrombectomy (4–7). Perfusion imaging allows estimation of salvageable brain tissue (penumbra) and tissue already infarcted or destined for infarction irrespective of reperfusion (ischemic core) (4, 7–11). Patient outcomes have been shown to be strongly related to the estimated volume of ischemic core at baseline (12, 13). As a result, CT perfusion (CTP) is increasingly being used in clinical practice around the world, with several software providing automated estimates of salvageable and ischemic core derived through various mathematical models (hemodynamic maps) (14, 15).

The hemodynamic maps generated by CTP are obtained by tracking a contrast medium as it flows into and out of the brain. The data is then processed using one of several different algorithms (14, 15). The estimation of salvageable tissue and ischemic core is then performed by applying a single threshold to one or two maps (9, 16, 17). However, there is significant variation between algorithms used when estimating tissue perfusion, and single-value thresholds have been shown to both under and overestimate the size of the infarct core and penumbra (18, 19). This may be partly due to the misclassification of image voxels as core or penumbra that results from single-value thresholding of core and penumbra. More sophisticated methods of processing CTP maps are required that can, for example, delineate artifactual signals from those caused by perfusion deficit.

The currently used perfusion thresholds have been validated to some degree and have shown success in selecting patients for treatment through clinical trials (6). However, a predictive model that uses all available perfusion data and spatial context of voxels may provide a more nuanced representation of the pathophysiology of evolving ischemic stroke, improving the accuracy of the images and the robustness of the output. Furthermore, shifting from a rigid single threshold model to a trained Machine Learning (ML) model is highly advantageous as the ML model may continue to improve performance with the addition of data.

There are many studies that develop and test ML and Deep Learning (DL) models for lesion segmentation and there have been great advances in developing applications of ML and DL to healthcare in general [e.g., (20, 21)]. However, there are challenges in widespread deployment such as lack of standardized methods to evaluate performance. Furthermore, the inner mathematical processes of ML and DL are often difficult to understand, and their outputs difficult to interpret. These issues of “explainability” and “interpretability” lead to ML being approached as a “black box” problem, without understanding of internal mechanisms. This has hampered implementation into medical practice. It is therefore essential to integrate ML in small, explainable steps rather than large, black-box overhauls that will result in issues of reliability (22). In this study we investigate if single-value thresholds for measurement of ischemic core and penumbra can be replaced with a ML-based method. We also outline challenges that must be addressed for successful integration into acute stroke assessment protocols.

## 2. Materials and methods

We developed an early ML model that is trained to delineate both ischemic core and penumbra from surrounding tissue using acute CTP data. We used retrospective data from an acute ischemic stroke patient cohort to develop models based on four ML algorithms (Logistic regression, Random Forest, XGBoost and Support Vector Machine). We tested performance of the model on an additional set of new, unseen patient data.

### 2.1. Data acquisition

We analyzed CTP images from the International Stroke Perfusion Imaging Registry (INSPIRE), which is a database of acute stroke perfusion imaging and associated clinical information. For this study we used consecutive patients presenting with acute ischemic stroke who had whole brain CTP and who were recruited into INSPIRE between 2010 and 2017 at the John Hunter Hospital, Newcastle, Australia. For standardization, only one site was used at this stage. As is routine in INSPIRE, patients all underwent baseline multimodal CT imaging with non-contrast CT, CTA, and CTP. Written informed consent was obtained from all participants, and the INSPIRE study was approved by the site's ethics committee (23).

To obtain the perfusion images, a total of 19 acquisitions occurred over 60 s. The CTP data were processed by commercial software MIStar (Apollo Medical Imaging Technology, Melbourne, VIC, Australia). CTP parameters were generated by applying the mathematical algorithm of singular value decomposition with delay and dispersion correction (24). The following four CTP parameters were generated: cerebral blood flow (CBF), cerebral blood volume (CBV), mean transit time (MTT), and delay time (DT). The penumbra and core volumes were defined with dual thresholds: DT at the threshold of 3 s for total ischemic lesion volume and CBF at the threshold setting of 30% for acute core volume (8, 16, 25). After single-value thresholding, core/penumbra areas were limited to a single lesion and artifactual or erroneous regions were removed. The resulting map was used as the ground truth (GT). Core/penumbra were reviewed by experts to ensure they were accurate.

To develop the model, we used 86 acute ischemic stroke patients with a large vessel occlusion (LVO): M1 segment of the middle cerebral artery (MCA) or internal carotid artery (ICA). To provide additional testing and external validation, 25 patients were used, with both LVO and non-LVO occlusions. This was done to observe whether a model trained only on lesions resulting from an occlusion of large vessel will perform as well when testing on a variety of occlusion sites. Each patient in the test set underwent follow-up MR diffusion-weighted imaging (DWI) between 24 and 72 h after onset. The volume (mL) of the infarct core, as estimated by MR-DWI, was recorded and used for external validation. On follow-up imaging, all patients had a thrombolysis in cerebral infarction (TICI) score of at least 2b, indicating relatively complete reperfusion of initially hypoperfused regions. In these cases, the volume of the acute CTP core should more closely match that of the follow-up infarct core and could therefore be used to validate the predictions.

### 2.2. Creating labeled data

#### 2.2.1. Class labels

**Figure 1:**
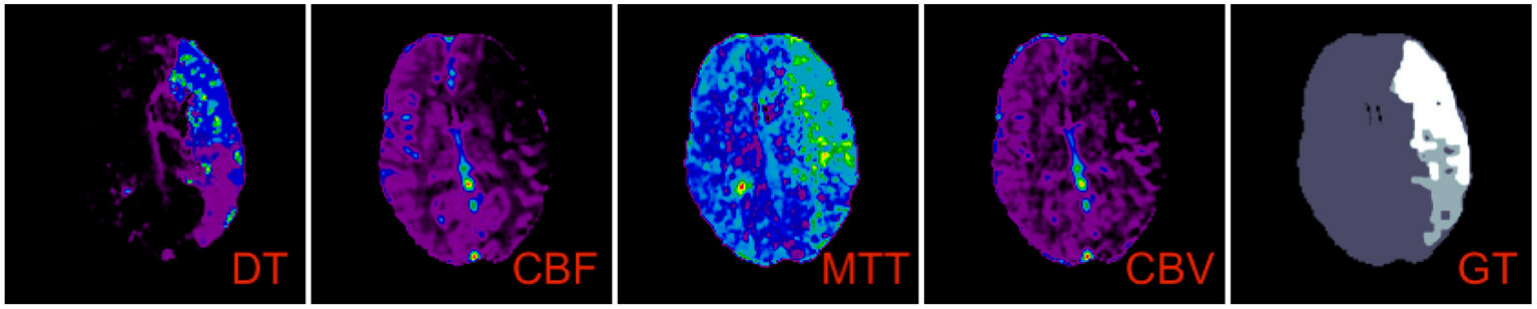
Feature maps and lesion map corresponding to the M1 test image. A single axial slice is shown with corresponding perfusion data for delay time (DT), cerebral blood flow (CBF), mean transit time (MTT) and cerebral blood volume (CBV). The corresponding class labels which make up the lesion map, used as ground truth (GT) in the algorithm, is shown on the far right.

The four hemodynamic maps (hereafter referred to as *features*) and core-penumbra segmentation maps (hereafter referred to as *lesion map*) were used in the development of the algorithm. The lesion map, together with the spatial coordinates of the mean baseline image from the CTP acquisition, was used to create a 3-D array of tissue class labels, where each voxel was one of four values: 0—background; 1—non-ischemic brain tissue; 2—penumbra; 3—core). Figure 1 shows the features alongside their class label array for a single patient.

#### 2.2.2. Under-sampling

For this early model, we avoided the issue of class imbalance by sampling the same number of voxels from each class in each image. We processed all lesion maps in the training data, counting the number of voxels belonging to each class. The smallest core volume contained 708 voxels and the smallest penumbra volume contained 8,436 voxels, and two images in the group had a penumbra but no core. We then randomly sampled 300 voxels from each class in each image. For the two images with no core, 300 extra healthy tissue samples were randomly taken from the image, ensuring 1,200 voxels were sampled from each feature channel.

#### 2.2.3. Patch analysis

**Figure 2:**
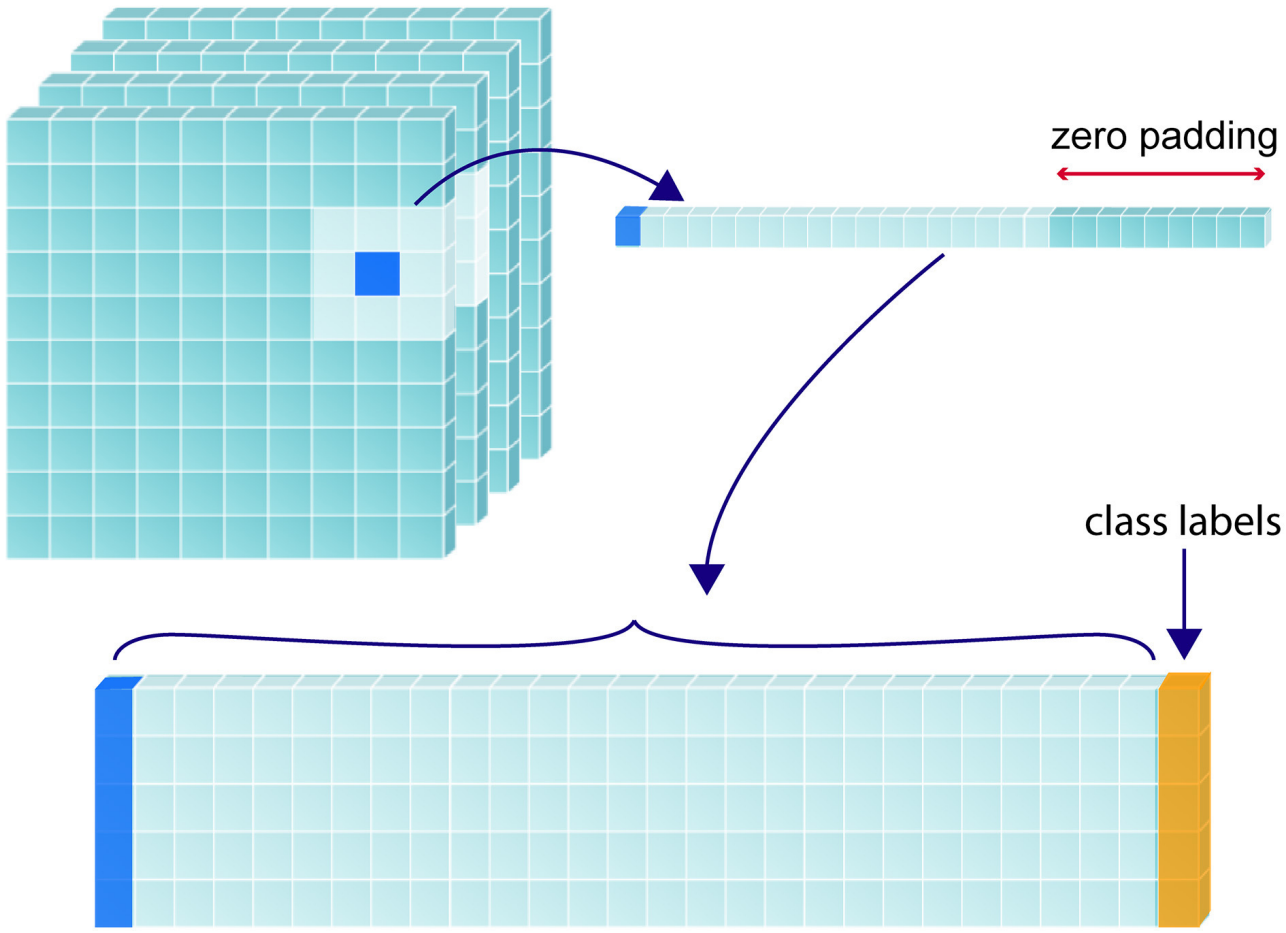
Construction of training matrix through sampling and patch extraction. For a given randomly selected sample (shown in dark blue), its corresponding perfusion map value/s and the values corresponding to its 26 immediate neighbors are collapsed into a 1-dimensional array, with the corresponding class label (yellow) added at the furthermost right position. If multiple perfusion maps are used, the 27 values from each map are recursively added to extend the 1-D array to the left of the class label. The 1-D array for each label are stacked to form a 2-D training matrix.

To predict the tissue status of a single sample (i.e., voxel of interest), we included the feature values associated with the coordinates of that voxel as well as the values associated with every direct neighboring voxel (26 in total), creating a patch-wise analysis. This was done to include spatial context in the determination of sample tissue status. Zero padding was used for samples that lay around the edges of the image. Figure 2 demonstrates this process for a single voxel of interest, where a 1-D array is created from the sample and its neighbors. Each sample resides in a single row of the training matrix, alongside its class label. All feature channels are concatenated along the same row.

### 2.3. Machine learning models

The sampled training data was further split into training (60%) and validation (40%) cohorts. Optimization and training were performed on the training data and evaluation was performed on the validation data. All data was standardized to [−1, 1] using the Standard Scalar function in Scikit-Learn in Python (v 0.0) (26).

We used Scikit-learn to optimize four models, based on logistic regression (LR), random forest (RF), XGBoost (XGB) and support vector machine (SVM), respectively. Except for SVM, a randomized search was initially performed, to estimate the best hyperparameters for each algorithm, after which a grid search was performed to narrow down the best hyperparameters. The chosen range for each hyperparameter was determined based on recommendations in Scikit-learn documentation. For each unique parameter combination, three-fold cross validation was performed.

### 2.4. Impact of added features

For this early model, we wished to determine whether performance was enhanced by including all four CTP maps vs. CBF and Delay Time alone. In particular, we wish to learn whether using four maps reduced the presence of artifactual perfusion lesions. Therefore, each model was trained twice; first with data only from CBF and Delay Time and then on data from CBF, Delay Time, MTT and CBV.

### 2.5. Performance evaluation

All the data used to train and optimize the model comprised random samples from images. However, the model will ultimately be used to process whole patient images and provide a prediction that can be displayed as an image. Therefore, we used an additional 25 whole brain patient images to further test the model's performance as it would be applied in a clinical scenario, and to provide a visualization of the model's accuracy. The images were processed as follows: from each voxel in the image, a 3D neighborhood patch was extracted and added to a matrix as in Figure 2. Each 3D patch from the image was forwarded through the model, and the resulting predictions were accumulated in a common space, preserving their spatial location and allowing the image to be reconstructed.

#### 2.5.1. Quantitative performance evaluation

The predictive model was trained using random samples, evenly distributed among the classes. For the test images, however, classes were severely imbalanced. Using receiver-operating characteristics (ROC) or average accuracy would favor the majority class and it is the minority classes that are of interest in this case. Furthermore, the area under the ROC curve (AUC) metric rewards positively predicted background pixels. Therefore, it is not a fair representation of the accuracy of a brain lesion segmentation, whereby background pixels constitute much of the image. For this reason, it was more appropriate to choose a metric more in line with perceptual quality, which reflects both size and localization agreement.

The Dice similarity coefficient (DSC) is a measure of spatial overlap for two regions (A, B), and is given by DSC (A, B) = 2(A ∩ B)/(A+B), where ∩ is the intersection. It can be seen as the percentage overlap between A and B. A perfect intersection between A and B will give a DSC of 1, and if there is no intersection between the two regions, the score is 0. DSC is sensitive to both size and location differences and is a highly intuitive manner of expressing similarity between two regions. We calculated the DSC between the ground truth and predicted images for the core and penumbra regions separately. After (27), DSC can be separated in a similar manner to the Kappa coefficient for agreement, into the following six categories (28, 29): 0, “No Agreement”, 0–0.2, “Slight agreement”; 0.2–0.4, “Fair agreement”; 0.4–0.6, “Moderate agreement”; 0.6–0.8; “Substantial agreement”; “0.8–1”; “Almost perfect agreement”.

The Jaccard Index (JI), also known as the Intersection of Union (IoU), like the DSC, ranges from 0 (no agreement) to 1 (perfect agreement). The JI is mathematically represented by IoU(A, B) = A ∩ B/A ∪ B, where ∪ is the union. The relationship between JI and DSC can therefore be described as JI = DSC /(2 − DSC). The DSC tends to be higher as it counts the true positive classifications twice in both the numerator and denominator of its equation, while the JI gives a greater penalty for bad classifications. Therefore, providing an average score over a set of classification will lead the average DSC and average JI to diverge from one another. The two metrics will always be positively correlated, however, we found it worthwhile to analyse the distinction as both are used throughout literature to evaluate segmentation tasks. The DSC and JI values for each the core and penumbra were calculated for all 25 images, and the differences between them were evaluated using paired *t*-tests.

Finally, lesion volume, one of the most important predictors of outcome after ischemic stroke, was calculated for the additional test images. The volumes of the core and penumbra were calculated for each of the ground truth and the predicted lesion by counting the number of voxels assigned to each area (30). Using pixel information encoded in the image, the absolute volume in milliliters could be calculated. As an external validation, the predicted core volume was compared with the follow-up (24–72h) infarct core derived from MR-DWI imaging and reviewed by the expert stroke neurologist (MP).

#### 2.5.2. Qualitative performance evaluation

**Figure 3:**
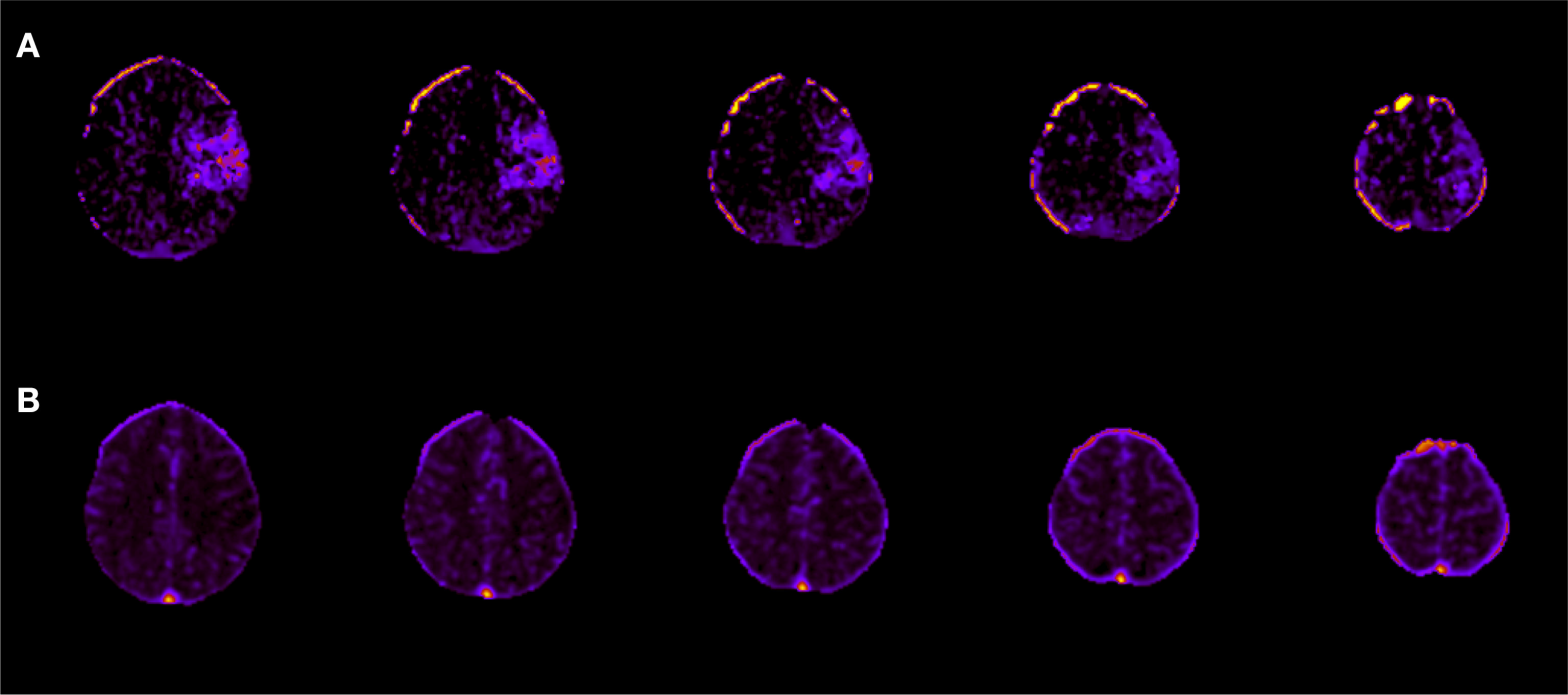
Skull artifacts. For subject 3, skull artifacts can be seen in their **(A)** Delay time and **(B)** CBF maps near the top of the skull.

We identified eleven images within the cohort affected by artifacts relating to the skull. In brain CT imaging, beam hardening from the dense skull region or, to a lesser degree, contrast-enhanced arteries, may result in a characteristic “streaking” artifact (31). When the skull, a highly attenuating region is adjacent to less attenuating tissue, such as soft tissue, and there is limited CT resolution, partial volume averaging may also occur. Here, the image intensity of affected voxels is a mixture, or an average, or the intensity of both these regions (32). Figure 3 shows an example of the partial volume artifact in Subject 3. Upon CTP processing, such voxels near the edge of the brain shows increased Delay Time. However, these artifacts are common and, if the image is otherwise of good quality, artifactual perfusion lesions are easy to identify to the trained eye. Therefore, we did not exclude these cases from the study and instead prefer to investigate the impact of artifact on model performance. We qualitatively compared the ability of the algorithms to make a correct prediction around those areas, based on both the inclusion of all four CTP maps and the additional spatial information provided by the 3D patches.

## 3. Results

For the training set, 55 patients had an occlusion of the M1 segment of the middle cerebral artery (MCA), and 31 had an occlusion of the internal carotid artery (ICA). Forty-three patients were female (50%), and the median onset age was 74 (IQR 63–82). The median baseline NIHSS (National Institutes of Health Stroke Scale) was 17 (IQR 14–20). Of these, 70 patients had a known time of onset; the median time between onset and CT imaging was 121 min (IQR 95–157). One patient had a wake-up stroke, and 15 patients had an unknown time of onset. Seventy-six patients received intravenous (IV) thrombolysis, one received intraarterial (IA) thrombectomy, two received both, five received no treatment and two patients did not have any treatment documented.

Three patients were discarded from the test set due to considerable infarct growth. For the remaining patients in the test set, 16 patients had an M2-MCA occlusion, four had an M3-MCA occlusion, and one each with an occlusion of the anterior cerebral artery (ACA) and ICA. Thirteen patients (59%) were female, and the median onset age was 79 (IQR 74–83). The median baseline NIHSS was 11 (IQR 6–16). In total, 20 patients had a known time of onset; the median time between onset and CT imaging for these patients was 110 min (IQR 96–168). The remaining patients had an unknown time of onset. Of all the patients in the test set, 20 received IV treatment, one received IA treatment and one received no treatment. Sixteen were given a TICI 3 score, and 6 were given a TICI 2b score. The median day of DWI image after stroke onset was 1 (IQR 1–2, min-max 0–12). The median size of the follow-up DWI core was as 10 mL (IQR 6–33). A Pearson correlation test shown a strong correlation (*p* < 0.005, two tailed) between the data used for the ground truth core measurement and the expert assessed MR-DWI measurements for core volume.

For model development, a total of 103,200 patch samples was used. Table 1 shows the class instances for the train and validation groups used to develop the model.

**Table 1:** Class representations across the training and validation cohorts.

	**Background**	**Non-ischemic brain**	**Core**	**Penumbra**
Train	15,485	15,870	15,141	15,424
Validation	10,315	10,530	10,059	10,376
Total	25,800	26,400	25,200	25,800

Samples were split into these categories using Scikit-Learn (26).

This was done to ensure the model did not bias any class.

Table 2 shows details of optimizing each model. Each model was trained using six computer processing units (CPU) in parallel. For SVM, only a random search for the two-map model was carried due to the excessive training times (>22 h), and only polynomial and linear kernels were tested, with the polynomial kernel outperforming the linear kernel. Table 3 shows results for each model on the under-sampled data. XGBoost was the highest performing algorithm, and there was an improvement in performance when all four CTP maps were included.

**Table 2:** Details of model training.

**Algorithm**	**^#^Parameters optimized**	**^#^Candidates in random search**	**Time taken (2 map, 4 map)**	**^#^Candidates in grid search**	**Time taken (2 map, 4 map)**
LR	6	28	5 min, 33 min	3	20 min, 53 min
RF	7	80	1 h 53 min, 3 h 1min	81	4 h 26 min, 6 58 min
XGB	5	10	30 min, 57 min	27	1 h 38 min, 2 h 53 min
SVM	3	30	22 h 33 min, N/A	N/A	N/A

Four different algorithms were used to train models: Logistic Regression (LR), Random Forest (RF), XGBoost (XGB) and Support Vector Machine (SVM). Each algorithm has different hyperparameters, and the number of different hyperparameters that were optimized here is shown (“^#^Parameters optimized”). Except for SVM, the parameters were optimized by first running a random search, training and testing models with a number of different random hyperparameter models (“^#^Candidates in random search”). The best performing combination was used to create the range for a more refined grid search. The number of candidates tested in the grid search was determined by the number of parameters that were optimized and the possible values for each parameter (e.g., whether values were discrete or continuous). The time that was taken to run all the different combinations is included. Six CPUs were used in parallel.

**Table 3:** Results of models on validation data.

	**ROC-AUC**	**DSC (core)**	**DSC (pen)**	**JI (core)**	**JI (pen)**
LR	0.9757	0.8438	0.7874	0.7298	0.6494
	0.9776	0.848	0.7907	0.736	0.6538
RF	0.9825	0.8553	0.8172	0.7471	0.6908
	0.9841	0.8611	0.8269	0.7561	0.7048
XGB	0.983	0.8552	0.8185	0.7470	0.6927
	**0.9844**	**0.8610**	**0.8275**	**0.7559**	**0.7057**
SVM	0.9799	0.8467	0.8081	0.7341	0.678

Eight models in total were optimized. Three algorithms (Logistic Regression/LR, Random Forest/RF, XGBoost/XGB) were trained twice, one on Cerebral Blood Flow (CBF) and Delay Time (DT) data (top), and once on data from CBF, DT, Cerebral Blood Flow and Mean Transit Time (bottom). Support Vector Machine (SVM) was only trained for two maps due to excessive training times. Results on the validation data are shown for each model. The highest performance across all categories was obtained for XBG, trained on all four CTP maps.

**Figure 4:**
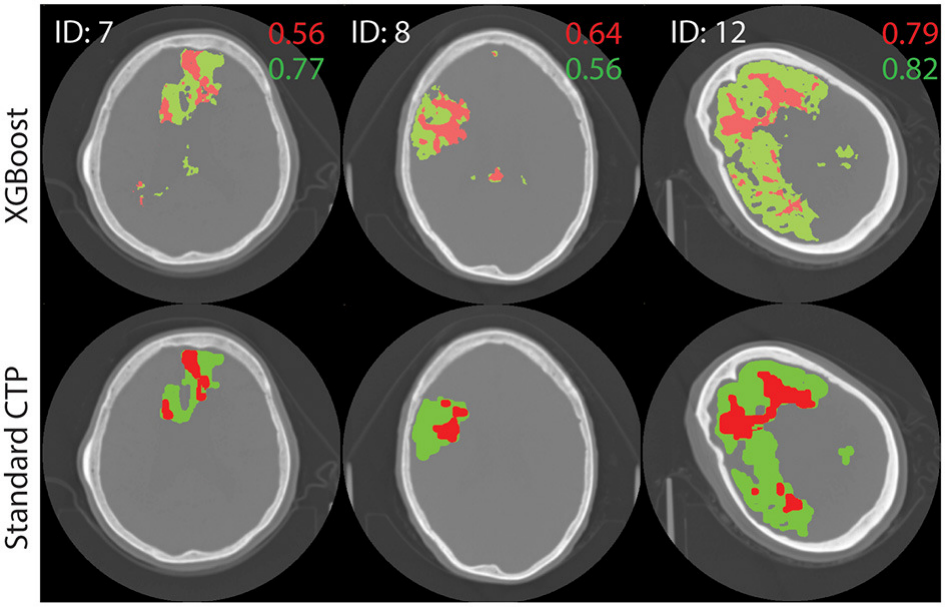
Test image results. A single axial slice, selected to clearly display the lesion, is shown from each image. The results of processing test images through the XGBoost model to make a prediction on the class label is shown at top. Standard lesion maps are shown at bottom. The predictions for core (red) and penumbra (green) are shown on top of a single axial slice of the brain, obtain with non-contrast CT. Dice similarity score are shown on the image, in corresponding colors.

The performance of the best performing model (shown in bold in Table 3) was tested on the remaining 22 images in the test set. The results are shown in Supplementary Table A1. Figure 4 shows axial slices of lesion predictions (overlayed on non-contrast CT image slices) using the model based on all four CTP maps for a selection of datasets (subjects 7, 8, and 1 with reference to Supplementary Table A1).

For all 22 patients, the mean DSC values for core and penumbra were 0.39 (SD 0.26) and 0.50 (SD 0.22), respectively, and the mean JI values for core and penumbra were 0.28 (SD 0.23) and 0.36 (SD 0.20), respectively. For both core and penumbra, JI and DSC were significantly different across the dataset (core: paired *t*-test, *p* < 0.0001; penumbra: paired *t*-test, *p* < 0.0001).

To explore the difference between performance on core and penumbra, a volume analysis was performed. Each similarity measured varied significantly with volume: A Pearson's correlation for DSC variation with volume showed (r = 0.56, *p* = 0.0065) for penumbra and (r = 0.71, *p* = 0.0002) for core. For JI a Pearson's correlation calculation showed (r = 0.61, *p* = 0.0028) for penumbra and (r = 0.72, *p* < 0.0002) for core.

Out of the 22 testing images, 16 lesions were due to an occlusion of the M2 segment of the MCA. The DSC scores for core and penumbra averaged to 0.34 (SD 0.23) and 0.50 ± 0.20, respectively. The mean volume of core and penumbra for M2 lesions was 9.89 mL (SD 8.17) and 38.34 mL (SD 22.5), respectively, lower as compared with the entire testing set.

**Figure 5:**
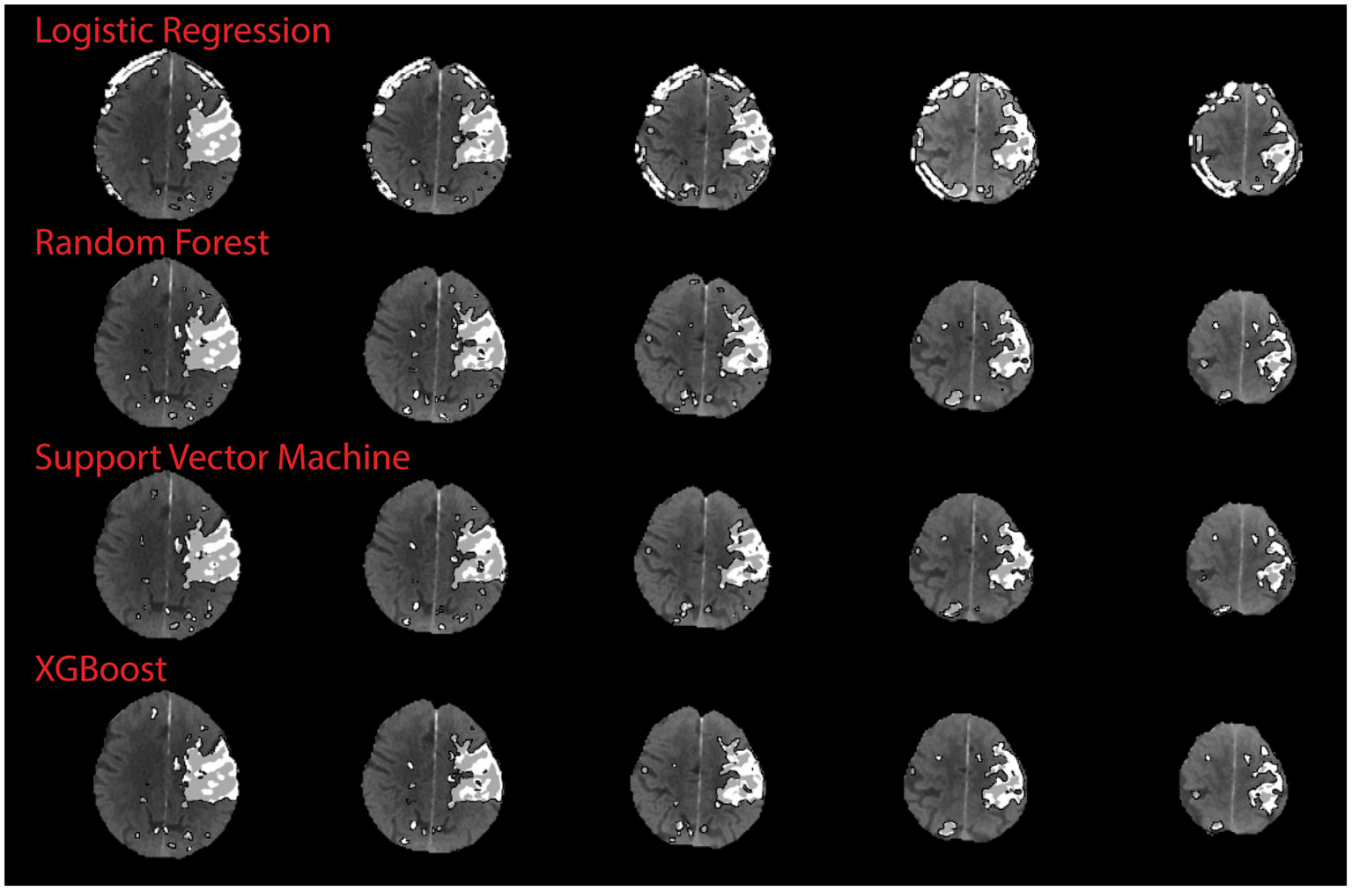
Qualitative results on skull artifact. Predictions made on subject 3 (shown in Figure 3) for each algorithm. Linear regression performed the poorest in terms of identifying artifact as areas of perfusion, as demonstration by subject 3 axial slices.

There was no significant correlation between the XGB-predicted core and the 24 h DWI infarct core (Pearson's r; r = 0.18, *p* = 0.41). However, visual inspection confirmed that artifacts due to the skull were present in half the cases (*n* = 11) and led to overestimation of perfusion regions. When considering the test cases with no obvious skull artifacts, there was a significant correlation between the predicted core and the follow-up DWI core (Pearson's r; r = 0.82, *p* = 0.0018). Figure 5 shows a comparison of results from each algorithm for the subject shown in Figure 3. This subject had significant CTP artifacts due to the skull. While LR could not distinguish actual from artifactual perfusion lesions, in this case all the other algorithms were able to.

## 4. Discussion

This study proposes a machine learning algorithm using the entire perfusion map datasets as an alternative to measuring the penumbra and ischemic core using binary thresholds with CTP data, Models based on four different well-known ML algorithms were tested. Accuracy was tested both quantitatively, using similarity measurements, and qualitatively, by using visual inspected to determine which algorithm was better at prediction on artifactual CTP hyperintensities. Simple neighborhood analysis was used to make a prediction on a single voxel; all surrounding voxels were considered. Our model may easily be expanded to include additional input channels, such as non-contrast CT, or relevant clinical information such as time-from-onset, blood pressure, clinical severity measurements and age.

Out of the four algorithms tested, XGBoost performed best in the quantitatively analysis, achieving good accuracy in mimicking the CTP perfusion lesions derived by the clinically used software MIStar. There was an improvement in performance when all four CTP maps were used compared to only CBF and DT for this early model. Future versions of the model will continue to use all four CTP maps to make a prediction.

Ideally, an automated CTP algorithm should differentiate between genuine and artifactual hypoperfusion patterns, just as an experienced stroke physician should be able to determine whether the pattern is topographically consistent with stroke phenotype (33, 34). For the qualitative study, SVM, XGB and RF improved on the ability of the LR algorithm to distinguish real from artifactual CTP hyperintensities. This is because LR is the only algorithm based on linear first-order interactions between variables, whereas the other three are more sophisticated, and able to model non-linear and higher interactions. This is shown clearly in Figure 5, although in other cases the ML model still derived artifact in making a prediction, leading to an overall worse correlation with the DWI infarct core for images with obvious artifact. As CT artifacts are difficult to avoid altogether in a clinical setting, this is a useful insight. Further development is required to ensure future versions of this model to not derive artifactual perfusion lesions.

For the testing images, DSC and JI scores were shown to vary significantly even though they are both commonly used similarity metrics. In addition, both metrics varied significantly with volume. Therefore, the DSC or JI score for a large lesion may not represent the same accuracy as for a small lesion, even though (27) has proposed otherwise (35, 36). For example, the large core in Figure 4 (ID = 12, M1) receives an almost perfect DSC value, while the smaller cores received lower DSC scores; these differences may have resulted merely from size differences. The same behavior was seen with JI. An average DSC or JI score that is a result of the summing over results from lesions of different sizes will not be an accurate representation of the overall performance of a model. We propose a weighted mean DSC/JI to account for size variation before these scores can be fully interpretable. Further studies will explore the application of a weighted mean. In lieu of a robust and subjective model performance metric, benchmark data [ISLES 2018 (37)] will be used in future studies to report performance.

The most significant limitation to this study is that, as a first step, we have used the CTP core and penumbra estimations derived by MIStar as the ground truth, even though these only approximate the ground truth. The gold standard in the determination of acute ischemic tissue is an expertly segmented MR-DWI lesion, either with (core) or without reperfusion (penumbra) (9, 38). Without a perfect ground truth, it remains difficult to interpret model performance in an objective fashion. For example, as MIStar maps are based on a simple thresholding method, a meaningful comparison of this ML method to a thresholding method against MIStar maps is challenging. Although MIStar and other software (39) CTP core estimates have been shown to be a fair approximation of DWI lesions previously, there are certainly ongoing issues (18), one of which is that the reference standard for core is imperfect (16). Nonetheless, future studies will adopt manually segmented DWI images as ground truth a therefore be able provide performance metrics that are more robust and interpretable. In addition, this model uses derived perfusion parameters rather than raw CTP time series images, risking a loss of valuable information contained in the raw images which may be lost in the derivation process. The model uses a simple approach over more advanced approaches that have been tested in the literature, such as those based on Deep Learning. With DL, features may be automatically extracted from images, both locally and globally, to make predictions with efficiency (40, 41). Future models will adopt DL, however, the current analysis using more explainable algorithms, was a necessary first step.

With this study we have shown that a Machine Learning method is capable of mimicking common use perfusion lesion measurements to a high accuracy. With the increasing prevalence of CTP assessments for treatment selection of ischemic stroke patients, particularly in the extended time window, it is vital that the measurements be accurate and representative of the underlying pathophysiology. There is significant scope for the current single threshold methods to overestimate the ischemic perfusion lesion and either under- or over-call the ischemic core depending on onset to reperfusion speed, and other factors. The proposed model may prove more accurate with further development than the currently used single threshold maps and can consider physiologically relevant information such as blood pressure, cardiac output and fluid status which would influence contrast flow and hence perfusion measures on the CTP. Imaging metadata such as time may also influence accuracy, as “ghost cores” have been noted in the hyperacute phase (42).

While the model is simple in its current form, we were able to demonstrate salient points about CTP-based predictions of stroke infarct. We have demonstrated that similarity indices such as DSC and JI have some difficulty in interpretations and further development of performance metrics is required. We have also demonstrated that non-linear algorithms are more adept at making predictions on common CT artifacts that linear model such as logistic regression. Further studies will use manually segmented DWI volumes as ground truth, as well as digest raw CTP data rather than post-processed CTP maps for Deep Learning predictions. Benchmark datasets will be used to measure performance. In addition, the role of clinical data and imaging metadata will be explored in making predictions.

## 5. Conclusion

We have described a Machine Learning model for the delineation of ischemic tissue from CTP data which is based on the XGBoost algorithm combined with 3D neighborhood analysis. The model is trained on lesion segmentations derived by clinically used software and can derive perfusion lesions to high accuracy. The model improves on clinically available software in that is it able to use multiple input channels but is currently limited by the lack of validation against gold standard lesion segmentations. Nonetheless, the model allowed us to demonstrate useful insight into CTP-based prediction of stroke infarct which will be used to make future developments.

## Data availability statement

The data analyzed in this study is subject to the following licenses/restrictions: De-identified data from the current study are available for qualified investigators upon reasonable request to the corresponding author. Requests to access these datasets should be directed to freda.werdiger@unimelb.edu.au.

## Author contributions

FW performed conception of the work, data analysis and interpretation, and drafting of the article. AB, MP, MV, TK, CL, NS, and LL provided critical revision of the article and final approval of the version to be published. All authors contributed to the article and approved the submitted version.
