# Supplementary material for: Machine learning segmentation of core and penumbra from acute stroke CT perfusion data
Source: Front Neurol. 2023 Feb 23;14:1098562. doi: 10.3389/fneur.2023.1098562 (PMC9995438; doi:10.3389/fneur.2023.1098562)
Supplement: Supplementary file 1 [file Table_1.DOCX]

Supplementary Material

| ID | Site | Treat† | 24hr DWI core | Pen. (mL) | Core (mL) | Pred. pen. (mL) | Pred. core (mL) | Acc. ^ŧ^ | Mean AUC ^ŧ^ | DSC (core) | DSC (pen) | JI (core) | JI (pen) |
| --- | --- | --- | --- | --- | --- | --- | --- | --- | --- | --- | --- | --- | --- |
| 1 | M2 | IV | 3 | 53.97 | 0.47 | 63.15 | 4.64 | 0.995 | 0.999 | 0.07 | 0.79 | 0.04 | 0.65 |
| 2* | M2 | None | 38 | 33.29 | 6.53 | 42.72 | 15.28 | 0.994 | 0.999 | 0.59 | 0.65 | 0.42 | 0.48 |
| 3 | M2 | IV | 17 | 30.32 | 14.5 | 106.51 | 52.34 | 0.979 | 0.991 | 0.43 | 0.23 | 0.27 | 0.13 |
| 4 | M2 | IV | 7 | 80.68 | 6.36 | 155.22 | 28.69 | 0.984 | 0.995 | 0.31 | 0.51 | 0.18 | 0.34 |
| 5 | M2 | IV | 1 | 36.62 | 15.4 | 74.29 | 131.2 | 0.974 | 0.994 | 0.21 | 0.26 | 0.12 | 0.15 |
| 6 | M2 | None | 4 | 8.62 | 0.50 | 14.51 | 5.53 | 0.997 | 1.000 | 0.15 | 0.54 | 0.08 | 0.37 |
| 7 | ACA | IV | 8 | 69.64 | 12.8 | 80.85 | 25.07 | 0.993 | 0.999 | 0.56 | 0.77 | 0.38 | 0.63 |
| 8 | M2 | IV | 27 | 25.09 | 11.5 | 33.34 | 23.24 | 0.995 | 0.999 | 0.64 | 0.56 | 0.47 | 0.39 |
| 9 | M3 | IV | 31 | 14.84 | 0.48 | 17.42 | 7.27 | 0.998 | 1.000 | 0.12 | 0.65 | 0.07 | 0.48 |
| 10 | M2 | IV | 45 | 19.05 | 30.9 | 80.63 | 38.78 | 0.989 | 0.995 | 0.87 | 0.30 | 0.77 | 0.18 |
| 11 | M1 | IV | 21 | 74.17 | 19.6 | 79.00 | 36.46 | 0.994 | 0.999 | 0.67 | 0.76 | 0.51 | 0.61 |
| 12 | M1 | IA | 35.6 | 220.31 | 88.0 | 267.62 | 74.97 | 0.988 | 0.998 | 0.79 | 0.82 | 0.65 | 0.70 |
| 13 | M2 | IV | 8 | 41.89 | 0.25 | 56.72 | 3.14 | 0.995 | 0.999 | 0.11 | 0.68 | 0.06 | 0.52 |
| 14 | M2 | IV | 28.1 | 23.12 | 18.1 | 30.73 | 22.52 | 0.996 | 0.999 | 0.78 | 0.65 | 0.64 | 0.48 |
| 15* | M3 | IV | 28 | 13.83 | 9.33 | 57.71 | 26.44 | 0.989 | 0.997 | 0.51 | 0.24 | 0.34 | 0.14 |
| 16 | M2 | IV | 3.1 | 61.20 | 21.6 | 97.94 | 60.53 | 0.987 | 0.997 | 0.50 | 0.61 | 0.34 | 0.44 |
| 17 | M2 | IV | 6.5 | 11.95 | 1.23 | 53.77 | 11.76 | 0.991 | 0.997 | 0.14 | 0.27 | 0.08 | 0.16 |
| 18 | M2 | IV | 18.1 | 37.50 | 21.2 | 107.14 | 43.75 | 0.984 | 0.995 | 0.44 | 0.42 | 0.28 | 0.27 |
| 19 | M2 | IV | 7.4 | 13.06 | 14.9 | 20.65 | 75.05 | 0.987 | 0.995 | 0.33 | 0.15 | 0.20 | 0.08 |
|  |  |  |  |  |  |  |  |  |  |  |  |  |  |
| 20* | Bas. | IV | 50 | 26.38 | 0.52 | 47.23 | 9.62 | 0.993 | 0.999 | 0.06 | 0.60 | 0.03 | 0.43 |
| 21 | M3 | IV | 1.5 | 10.90 | 0.10 | 16.18 | 12.54 | 0.996 | 0.999 | 0.02 | 0.41 | 0.01 | 0.26 |
| 22 | M2 | IV | 9.1 | 7.12 | 2.66 | 78.54 | 15.06 | 0.982 | 0.996 | 0.27 | 0.11 | 0.16 | 0.06 |
| 23 | M2 | IV | 32.5 | 40.38 | 8.05 | 66.55 | 34.37 | 0.990 | 0.998 | 0.37 | 0.56 | 0.23 | 0.39 |
| 24 | M3 | IV | 60 | 92.68 | 43.1 | 104.38 | 84.36 | 0.988 | 0.998 | 0.66 | 0.69 | 0.49 | 0.53 |
| 25 | M2 | IV | 3 | 60.11 | 3.57 | 188.05 | 82.12 | 0.972 | 0.994 | 0.08 | 0.33 | 0.04 | 0.20 |

*Subjects experienced significant infarct growth and were excluded from the study.

† Intravenous thrombolysis (IV); intra-arterial thrombectomy (IA)

Ŧ Background pixels are included in accuracy and AUC calculations leading to high numbers

**Supplementary Table 1.** Results for the 25 testing images, with three exclusions. The location of the thrombus associated with each lesion is included (‘site’), as is the treatment path for each patient (‘Treat’). Volume according to standard CTP (‘Core (mL)’ and ‘Pen. (mL)’) is included alongside volumes predicted by the ML model (‘Pred. core (mL)’ and ‘Pred. penumbra (mL)’). The volume of the infarct core estimated by follow up (24-72hr) DWI images associated with each patient is included (‘24h DWI core (mL)’) is included alone with all accuracy metrics described in the article.
